# Supplementary figures and images for: Lumenal calcification and microvasculopathy in fetuin-A-deficient mice lead to multiple organ morbidity
Source: PLoS One. 2020 Feb 19;15(2):e0228503. doi: 10.1371/journal.pone.0228503 (PMC7029858; doi:10.1371/journal.pone.0228503)

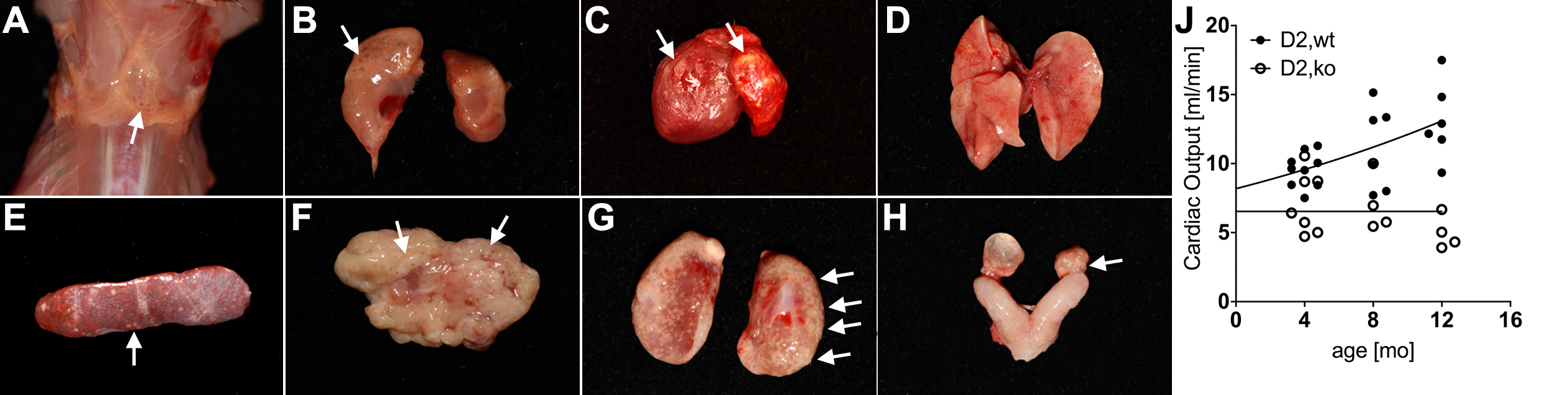

Supplement: S1 Fig — In unstained tissues, the nodular calcified lesions appear off-whitish, semi-transparent granules of sub-millimeter size. Nodules can be harvested mechanically by scraping of (A) brown adipose tissue in the neck, or from the subcutaneous fat layer of the skin (see main-text Fig 6A–6C). Nodules are present in the kidney fat pads (B), but not the kidney pelvis, of 6-week-old a D2,Ahsg-/- mice, ventricular wall and the atrium (C), lung tissue (D), spleen (E), pancreas (F), 16-week old kidney pelvis (G), and ovaries (H). J, Echocardiography showed that cardiac output was reduced in fetuin-A-deficient DBA/2 mice compared to wildtype mice at all ages measured. (JPG) [file pone.0228503.s001.jpg]

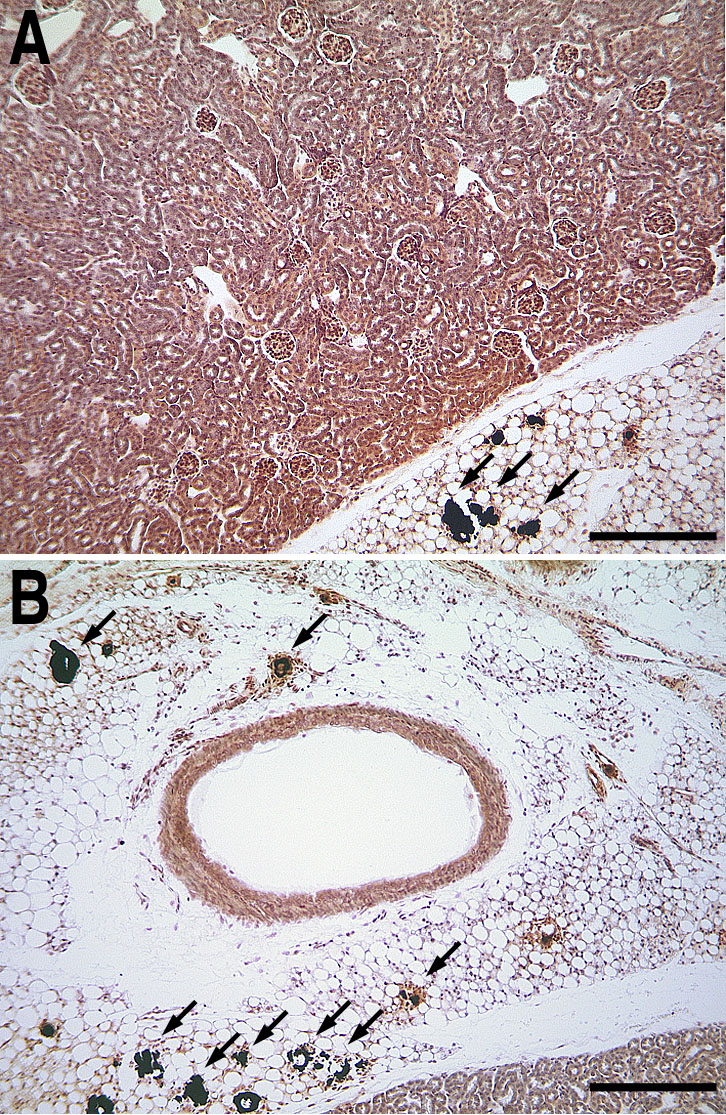

Supplement: S2 Fig — Representative von Kossa staining for mineral in paraffin sections of kidney (A) and brown adipose tissue (B) from a 6-week-old D2,Ahsg-/- mouse. Calcified lesions, indicated by black staining (arrows) can be observed in brown adipose tissue but not in the kidney parenchyma, reflecting the different calcification state of both tissues. Scale bars 200 μm. (JPG) [file pone.0228503.s002.jpg]

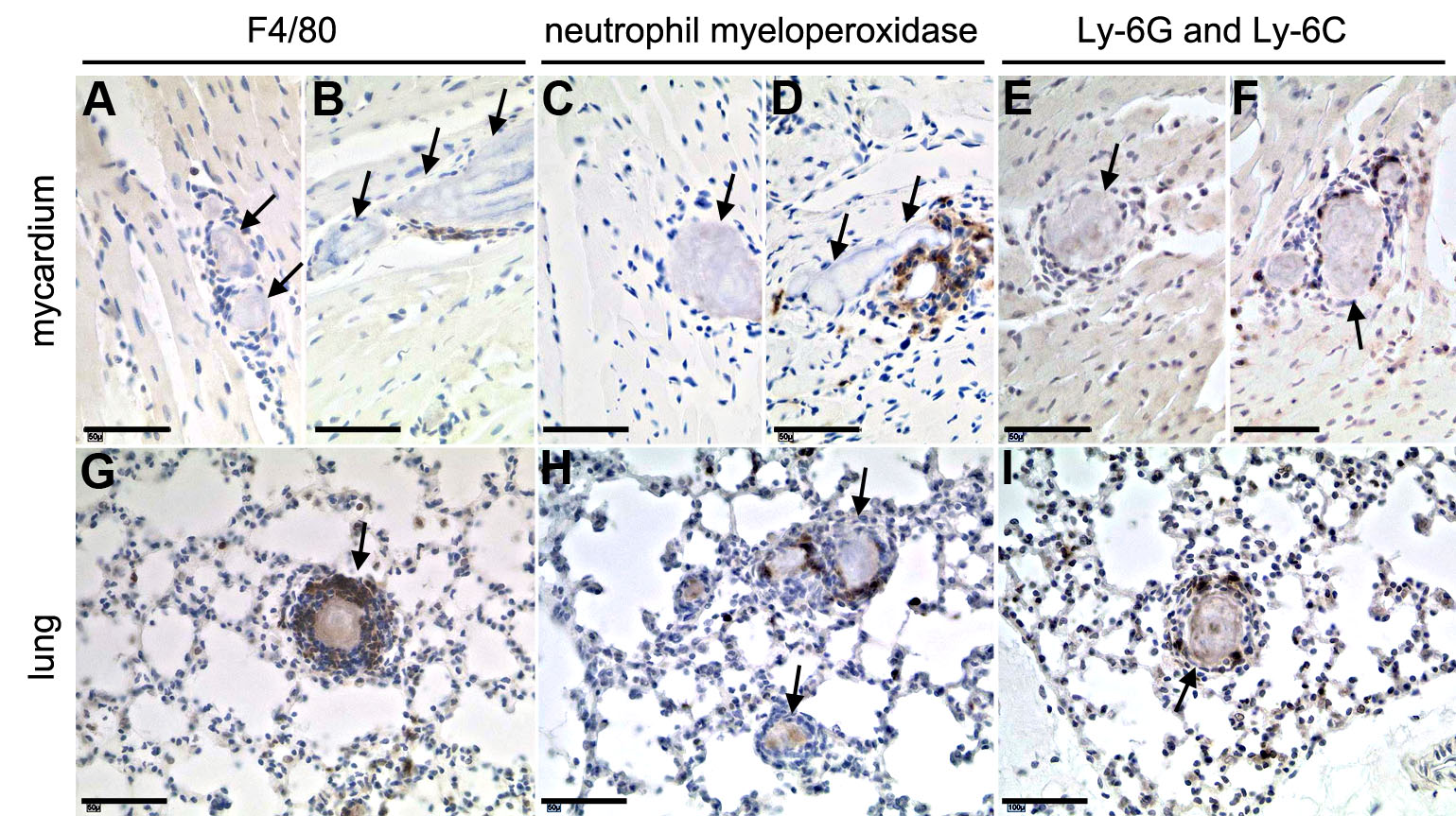

Supplement: S3 Fig — Paraffin sections of 11-week-old D2,Ahsg-/- mice were prepared from myocardium (A-F) or lung (G-I) tissue. Sections were stained with antibodies against the macrophage surface marker F4/80 (A, B, G), neutrophil myeloperoxidase (C, D, H) and the monocyte/granulocyte marker Ly-6G and Ly-6C (Gr-1). Scale bars 50 μm. (JPG) [file pone.0228503.s003.jpg]
