# Supplementary material for: Lumenal calcification and microvasculopathy in fetuin-A-deficient mice lead to multiple organ morbidity
Source: PLoS One. 2020 Feb 19;15(2):e0228503. doi: 10.1371/journal.pone.0228503 (PMC7029858; doi:10.1371/journal.pone.0228503)
Supplement: Attachment — Submitted filename: 20191223_Response to Reviewers.docx [file pone.0228503.s012.docx]

General Comments

We were asked by PLoS ONE to provide additional information on the data provided in figure 8 and Figure S1 of the manuscript. We added this text to the methods section:

Our answer: The survival analysis shown in Figure 8 was carried out retrospectively based on routinely collected data in the period dating from 2007-2008. Breeding of DBA/2, Ahsg -/- homozygous mice was stopped after the higher death rate for fetuin-A deficieny in the DBA/2 background was realized. The strain is now maintained symptom-free as heterozygotes. Survival monitoring data were obtained on the daily basis from birth until natural death and documented in the institutional animal database as well as in records of laboratory personnel and qualified animal caretakers on corresponding animal ID cards. None of included animals had been euthanized. Trained and experienced animal caretakers and investigators have not detected any abnormalities in observed animals regarding their ability to move, access food and water, respiratory changes, body changes. Life span (age in days at the timepoint of natural death) data had been extracted from the total pool of the animals free of any kind of chemical or other interventions (e.g. used as control) separately for each genotype. Premature death was not accompanied with detectable suffering, animals stayed active and though they show slower movements they still reached food and water, had no wounds, nor respiratory difficulties.

We now include the underlying data for Figures 8 and Figure S1 J in a file called “STable 1 Mouse Data”

Also we added statements regarding the deposition of raw genomic and proteomic data.

Our answer: Raw gene chip data were submitted according to MIAME format to gene expression omnibus GEO (GSE140954).

Raw mass spectrometry data is deposited in PeptideAtlas at: http://www.peptideatlas.org/PASS/PASS00920.

Review Comments to the Author

Reviewer #1

Summary:

Herrmann et al present a manuscript describing the phenotype the Fetuin-A (Ahsg) knock out mouse on the DBA/2 background (D2,Ahsg-ko) using microCT, electron microscopy, transcriptional and proteomic analysis. Major finding stated are the reduced growth and premature death of these mice as compared to strain-matched controls due to extensive ectopic calcification of the soft tissues. The authors conclude that calcification in these mice initiates in the lumen microvasculature due to the absence of the fetuin-A mediated solubilization and clearance of excess calcium-phosphate from the circulation.

Comments:

It appears that females are strikingly more sensitive to an absence of Fetuin-A than their male counterparts, but this was not addressed at any length, other than mentioning this difference. Do WT females have more circulating feutin-A at baseline? Are women more prove to CKD-related calcification than men? Do they have less fetuin-A? Was the cardiac output shown in S1 in males or females? This information would be more informative if output was also graphed by gender. The cause of death was stated as “most likely” due to cardiac failure, yet, there was no histology (for example, picrosirius red, masson trichrome) supporting this. Additionally, as the pancreas was severely calcified, how is the glucose tolerance of these mice?

Our answer: We thank Reviewer 1 for his/her comments. We now realize that the presentation of results in chronological, instead of logical order, was the more confusing option. We now first inform about the major results of this study; namely, that calcification starts in the fluid phase of small capillaries and involves coagulum/protein-rich deposits that likely reflect plasma coagulated onto a mineral-rich contact phase. To emphasize this crucial result we rearranged the data presentation order starting with previous Figure 2 (now Figure 1). We now show the original Figure 1 at the end of the Results section. The data are still important, because they illustrate late consequences of intraluminal calcification, yet unfortunately they do not add mechanistic insight into the origin of calcification. We present these historic population-based data from our colony because they are crucial for comparing calcifying mouse strains and their underlying pathologies. Rarely are such data published, because they take years to collect.

Regarding the sexual dimorphism in D2,Ahsg-/-mice, we emphasize that hemizygous D2, Ahsg +/- do not develop the strong calcification phenotype demonstrating that half the baseline level of fetuin-A protein is enough to prevent widespread calcification and the ensuing pathologies. Fetuin-A levels at baseline are essentially similar in both sexes, certainly with this fifty percent margin. We would like to stress once more, that the strong calcification phenotype of the Ahsg knockout mice only appears on the DBA/2 background, and that these mice have a compound phenotype of complete fetuin-A deficiency, as well as defects in handling two more regulators of extracellular mineral homeostasis, Mg and pyrophosphate, which we described in an accompanying paper (Babler et al bioRxiv. doi: 10.1101/577239). The point we try to make in this manuscript is that fetuin-A, Mg and pyrophosphate safeguard against unstable CPP formation, and ultimately against the spontaneous formation of what would be disastrous crystalline mineral in the fluid phase of the blood, something never demonstrated before. To emphasize this point we changed the title to “Luminal Calcification and Microvasculopathy in Fetuin-A-Deficient Mice Lead to Multiple Organ Morbidity”.

We regard the sexual dimorphism as a consequence of sex-specific organ failure secondary to calcification. This is also supported by our gene expression analysis, where sex specific differences where considered in the analysis, yet they did not reveal any differences. There are several reasons why females may react different than males. The survival curves shown in Fig. 8D (was 1D) contain homozygous female breeders, which generally stop breeding after one or two pregnancies and show extensive calcification of their reproductive organs. We hypothesize that pregnancy and lactation, with the ensuing mobilization and transport of mineral to meet the needs of fetuses and pups, put extra stress on the extracellular mineral ion-buffering system of fetuin A-mediated CPP formation and that this may contribute to the higher female lethality. In fact, we reported in the original description of Ahsg-knockout mice on a mixed C57BL/6 X Sv129 background that spontaneous ectopic calcification appeared only in female ex-breeders (Jahnen-Dechent, W., Schinke, T., Trindl, A., Müller-Esterl, W., Sablitzky, F., Kaiser, S., and Blessing M (1997). Cloning and targeted deletion of the mouse fetuin gene. J Biol Chem 272, 31496–31503.) We now mention this at the start of the discussion.

The D2,Ahsg-/- mice die suddenly and are usually found dead in the morning. The most logical explanation is swift cardiovascular death - infarction or stroke. In one of our previous studies we in fact demonstrated that the mice have severely compromised cardiovascular health supporting this view (Merx, M.W., Schäfer, C., Westenfeld, R., Brandenburg, V., Hidajat, S., Weber, C., Ketteler, M., and Jahnen-Dechent, W. (2005). Myocardial stiffness, cardiac remodeling, and diastolic dysfunction in calcification-prone fetuin-A-deficient mice. J Am Soc Nephrol 16, 3357–3364). Histology, including picrosirius red staining as requested by Reviewer 1, and indeed Langendorff-type functional heart studies, as well as echocardiography, were all employed in this previous study.

We state in the discussion that the calcification starts inside the lumen of the vasculature primarily affecting highly capillarized organs. To our knowledge a similar phenotype has not been reported in any other calcifying mouse phenotype. In search of the initial trigger for calcification we have in the past studied several organs in isolation, and reported what are the consequences of multiple-organ calcifications:

- secondary hyperparathyrodism and albuminurea secondary to kidney damage (Schäfer, C., Heiss, A., Schwarz, A., Westenfeld, R., Ketteler, M., Floege, J., Müller-Esterl, W., Schinke, T., and Jahnen-Dechent, W. (2003). The serum protein alpha 2-Heremans-Schmid glycoprotein/fetuin-A is a systemically acting inhibitor of ectopic calcification. J Clin Invest 112, 357–366.),

-bone formation defects associated with growth plate capillary abnormalities

Brylka, L.J., Koeppert, S., Babler, A., Kratz, B., Denecke, B., Yorgan, T.A., Etich, J., Costa, I.G., Brachvogel, B., Boor, P., et al. (2017). Post-weaning epiphysiolysis causes distal femur dysplasia and foreshortened hindlimbs in fetuin-A-deficient mice. PLoS ONE 12, e0187030.

- myocardial stiffness, cardiac remodeling, and diastolic dysfunction (Merx et al. (2005) cited above.

Studying glucose tolerance secondary to pancreas calcification as suggested by the reviewer is something that would surely add to these studies, but it will not help in identifying the initial trigger of calcification, which was the aim of the current study. We respectfully maintain that the glucose work falls outside the main calcification discourse of our paper.

The authors make the strong statement that calcification is not associated with osteogenic differentiation. This statement is based on genome wide expression analysis of the kidney and brown fat and the data does not show increased expression of traditional makers of osteogenic differentiation. This conclusion is an overstatement. The several pre-ceding figures clearly shows that calcification initiates in the microvasculature, yet this transcriptional analysis was performed on whole organ tissue, not on microvascular cells. The precise cells involved in microvascular mineralization (ECs, SMCs) should be used for this sort of analysis. In the absence of collecting these cells in vivo, lines from WT and D2,Ahsg-ko cells could be made. This statement “seems that osteochondral cell differentiation is consequence, rather than a cause, of mineralization in soft tissues“, appears to apply this argument to all forms of ectopic calcification; this is not at all supported by the data in this study and should be rephrased.

Our answer: The tissues we studied did in fact also contain the microvascular cells mentioned by the reviewer, but they were analyzed in bulk with other cells in these organs. Theoretically one could analyze these cells for osteochondrogenic differentiation by single cell sequencing if one were quick enough to be able to harvest the cells before large calcified lesions are formed. However, this would again mean an analysis of damaged cells secondary to the calcification event, which is primarily an issue of solubility and stability of protein-mineral complexes, not an issue of gene induction. This, and the fact that in histology including EM we never saw any osteochondrogenic cells (unlike e.g. the copious “fish eye cell” chondrocytes in atherosclerotic lesions in Apo E-knockout mice), supports our strong statement. The pathway analysis of proteins expressed in the lesions, which we mention in the results likewise did not indicate osteochondrogenic differentiation. Therefore we stand by our statement that in D2,Ahsg-ko mice calcification is not associated with osteogenic differentiation, a statement also supported by comments made by Reviewer 2. If required, we gladly agree to elaborate on this in the Discussion section, and to publish the full discussion of this review process along with the paper, should it be accepted. Ref 57 summarizes a parallel discussion in the soft tissue calcification field that mineralization events can precede osteochondrogenic differentiation.

Minor Comments:

The symbols in Fig1 are difficult to discern. They should be made larger or color coded.

Our answer: Changed with color coding, as suggested.

The proteomic data in table1-3 could be better organized/grouped together, and more importantly, what are some of the highly up/down regulated proteins?

Our answer: The proteomic analysis in this study was meant to provide insights into the proteins contained in calcified lesions and most importantly to investigate whether typical bone matrix proteins would be contained in lesions. This was performed in several organs, namely adipose tissue, skin and heart, corresponding to the tissues where severe lesions were readily detected, enabling us to dissect and prepare for mass spectrometry. The experiment was not designed to provide quantitative data on differential protein regulation. Therefore, we did not present up/down regulated proteins but rather tried to categorize the proteins, which were specifically detected in calcified lesions but not the corresponding surrounding tissue, using pathway analyses tools. We feel that this format is more meaningful to what we are trying to show. While this data provides only a qualitative insight into the protein content in calcified lesions, it clearly demonstrates that lesions in several different tissues do not contain any typical compounds of the bone matrix, supporting our hypothesis that calcification in D2,Ahsg-ko mice is not associated with osteogenic differentiation.

The data from the gene expression array and the proteomic analysis must be put on a publicly available server, per PLOS ONE rules.

Our answer: The proteomic data have now been deposited in PeptideAtlas at:

http://www.peptideatlas.org/PASS/PASS00920.

The genomic data are deposited at GEO in MIAME format (GEO Number: GSE140954)

Reviewer #2

This is a really important manuscript for the study of cardiovascular calcification in general. The manuscript presents an animal model that is prone to vascular calcification and evaluate the origins and characteristics of the protein-mineral particles formed in the vasculature and their relation to Fetuin-A.

As said, this is an important addition to the subject, but during the discussion of the manuscript the text ignores several works on the literature that indeed support several of the claims presented in the work.

Cardiovascular calcification has been extensively characterised in the literature and for example the work of Bertazzo have demonstrated that the calcification present in the vascular tissue present different physico-chemical characteristics to bone, what supports the conclusions presented in this manuscript. Moreover, there is also other works by Hutcheson, that also present characterisation and studies about possible origin for vascular calcification that is similar to the calcification presented by the authors, again supporting several of their conclusions.

Therefore, the results presented in the mentioned works should also be discussed in the manuscript what would help readers to have a better idea of the existing (and sometimes competing) models and ideas about vascular calcification.

Our answer: Thank you for the comments about the importance of our work. We have now included references by Bertazzo et al and by Hutcheson et al as suggested by the reviewer.

Finally, would be good to have some scanning electron micrographs of the calcification and not only transmission micrographs, since the SEM would present a good idea of the 3D morphology of the calcification and even would help to understand the similarities and differences between the calcification presented by the authors and previous characterisation of human vascular calcification.

Our answer: Initially we employed high-sensitivity detection of calcification sites by immunofluorescence microscopy for the mineral-binding proteins osteopontin and fetuin-A, which allowed early detection of very small mineralized lesions at 2 weeks of age that could not be readily detected by routine histology or computed tomography. After this, only then were we able to prepare early-lesion tissues for high-resolution TEM/SAED/XRD/EDS. We indeed agree with the reviewer that additional results could have been obtained with SEM, but we have not prepared any tissue samples for this analysis, and opted for the higher resolution of the methods that we used. We maintain that we were able to get the results we needed using the methods we applied.
